# A novel balance training approach: Biomechanical study of virtual reality-based skateboarding

**DOI:** 10.3389/fbioe.2023.1136368

**Published:** 2023-02-10

**Authors:** Phunsuk Kantha, Wei-Li Hsu, Po-Jung Chen, Yi-Ching Tsai, Jiu-Jenq Lin

**Affiliations:** ^1^ School and Graduate Institute of Physical Therapy, College of Medicine, National Taiwan University, Taipei, Taiwan; ^2^ Physical Therapy Center, National Taiwan University Hospital, Taipei, Taiwan; ^3^ Division of Physical Therapy, Department of Physical Medicine and Rehabilitation, National Taiwan University Hospital, Taipei, Taiwan

**Keywords:** balance, virtual reality, skateboarding, biomechanics, training

## Abstract

**Introduction:** The use of virtual reality (VR) technology in training and rehabilitation gained increasing attention in recent years due to its potential to provide immersive and interactive experiences. We developed a novel VR-based balance training, VR-skateboarding, for improving balance. It is important to investigate the biomechanical aspects of this training, as it would have benefited both health professionals and software engineers.

**Aims:** This study aimed to compare the biomechanical characteristics of VR-skateboarding with those of walking.

**Materials and Methods:** Twenty young participants (10 males and 10 females) were recruited. Participants underwent VR-skateboarding and walking at the comfortable walking speed, with the treadmill set at the same speed for both tasks. The motion capture system and electromyography were used to determine joint kinematics and muscle activity of the trunk and legs, respectively. The force platform was also used to collect the ground reaction force.

**Results:** Participants demonstrated increased trunk flexion angles and muscle activity of trunk extensor during VR-skateboarding than during walking (*p* < 0.01). For the supporting leg, participants’ joint angles of hip flexion and ankle dorsiflexion, as well as muscle activity of knee extensor, were higher during VR-skateboarding than during walking (*p* < 0.01). For the moving leg, only hip flexion increased in VR-skateboarding when compared to walking (*p* < 0.01). Furthermore, participants increased weight distribution in the supporting leg during VR-skateboarding (*p* < 0.01).

**Conclusion:** VR-skateboarding is a novel VR-based balance training that has been found to improve balance through increased trunk and hip flexion, facilitated knee extensor muscles, and increased weight distribution on the supporting leg compared to walking. These differences in biomechanical characteristics have potential clinical implications for both health professionals and software engineers. Health professionals may consider incorporating VR-skateboarding into training protocols to improve balance, while software engineers may use this information to design new features in VR systems. Our study suggests that the impact of VR-skateboarding particularly manifest when focusing on the supporting leg.

## Introduction

Virtual reality (VR) technology allows users to interact with computer-generated environments in a simulated environment (Cipresso et al., 2018). In healthcare, VR has been used as a tool for rehabilitation and training, with the potential to improve motor function, cognitive function, and psychological wellbeing in individuals with various conditions, such as stroke, low back pain, and Parkinson’s disease (Lheureux et al., 2020; Liang et al., 2022; Yalfani et al., 2022). The immersive nature of VR can increase adherence and motivation to training programs and lead to improved outcomes (Moon et al., 2021; Recenti et al., 2021). Moreover, VR can be used for sensory integration exercise as it engages multiple senses, including vestibular, vision, and proprioception, simultaneously (Yen et al., 2011). Thus, studies have found that VR-based training can be effective in improving motor function, balance, and mobility in individuals who have had a stroke, as well as cognitive function in those with brain injury and other neurological conditions (Kumar et al., 2018; Chen et al., 2021). VR-based training has also been shown to have positive effects on psychological well-being, such as reducing anxiety and depression in individuals with chronic pain (Rawlins et al., 2021). Therefore, VR-based training has the potential to enhance the effectiveness of various interventions in healthcare.

Exergames, also known as exercise games, are interactive technology-based physical activities that are designed to provide an enjoyable and engaging way to get physically active (Sween et al., 2014). Exergames have gained popularity in recent years, particularly among older adults or individuals with chronic conditions, as a way to promote physical activity and improve physical fitness (Sween et al., 2014; Moret et al., 2022). Exergames can involve a wide range of physical activities, from dancing and jumping to moving arms and using other body movements to control the game (Asín-Prieto et al., 2020; Lopes et al., 2020). Research has shown that regular participation in exergames can improve coordination, balance, and other physical fitness measures, as well as reduce stress and improve mental health outcomes such as mood and cognitive function (Sween et al., 2014; Moret et al., 2022). Exergames can be played on video game consoles, smartphones, and VR head-mounted displays, and are suitable for people of all ages and fitness levels (Asín-Prieto et al., 2020). The evidence on the effects of exergames on health outcomes is mixed, overall, they suggest that exergames can be a useful tool for promoting physical activity and improving physical and mental health.

Unilateral leg training is a type of exercise that focuses on strengthening and conditioning one leg at a time (Liao et al., 2022). This type of training can be useful for a variety of purposes, including improving muscle imbalances, preventing injuries, and rehabilitating after an injury (Manca et al., 2017; Liao et al., 2022). Unilateral leg training can be performed using a variety of exercises, such as lunges, single-leg squats, and single-leg deadlifts, using body weight or added resistance (Baumgart et al., 2017; Manca et al., 2017). This type of training can be especially beneficial for athletes and individuals with a history of lower body injuries, as it can help to improve balance, stability, and overall leg strength (Liao et al., 2022). In addition, research has shown that unilateral leg training can be effective for improving muscle strength and power, as well as increasing muscle activation and coordination (Zhou et al., 2022). Unilateral leg training can be incorporated into a well-rounded fitness routine along with other forms of exercise to improve overall physical fitness and balance performance (Manca et al., 2017; Liao et al., 2022). However, it is important to use proper technique to prevent injury and ensure optimal results.

In order to combine VR-based training, exergames, and unilateral leg training, we developed an exergame called virtual reality skateboarding (VR-skateboarding). Moreover, VR technology was used to simulate a real-world environment in a safe setting, as well as to provide task-specific training for balance in the unilateral leg. However, the biomechanical characteristics of VR-skateboarding have not yet been fully explored. Therefore, we conducted a study to compare the biomechanical characteristics of VR-skateboarding with those of walking. We chose to compare these two activities because they involve similar movement patterns, such as repetitive leg movements. We hypothesized that VR-skateboarding would result in greater joint angles, muscle activity, and weight distribution compared to walking, which could potentially improve balance. The findings of this study could be useful for health professionals in understanding the mechanisms of training effects and designing training protocols, as well as for software engineers in creating new features and implementing multidisciplinary approaches.

## Materials and methods

### VR-skateboarding

**FIGURE 1:**
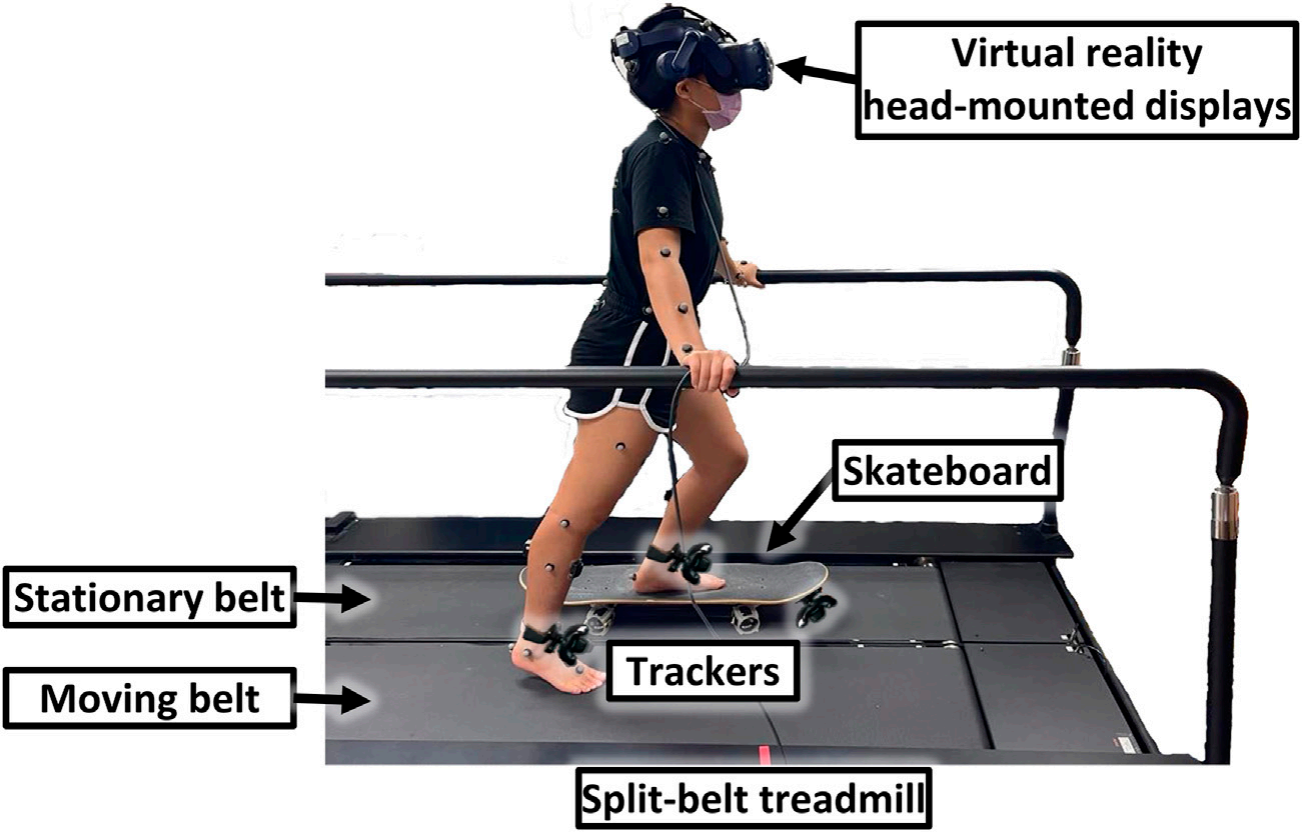
An illustration of a virtual reality skateboarding system.

VR-skateboarding is a training approach that combines VR and treadmill technology. It involved using a skateboard that was integrated with a split-belt treadmill (QQ-mill, Motekforce Link, Netherlands), as shown in Figure 1; Supplementary file S1. The skateboard was placed on the stationary belt of the treadmill, while the moving belt was set to a comfortable walking speed for the participant. Comfortable walking speed was measured using a 10-m walk test, which is the most common and reliable test (Bohannon, 1997; Bohannon and Williams Andrews, 2011; Cheng et al., 2020b). The leg that was placed on the skateboard was referred to as the “supporting leg,” while the leg that slide on the moving belt was referred to as the “moving leg.” For safety purposes, the skateboard wheels were fixed statically on the stationary belt of the treadmill. Handrails were also available at waist level for support during VR-skateboarding, if needed.

**FIGURE 2:**
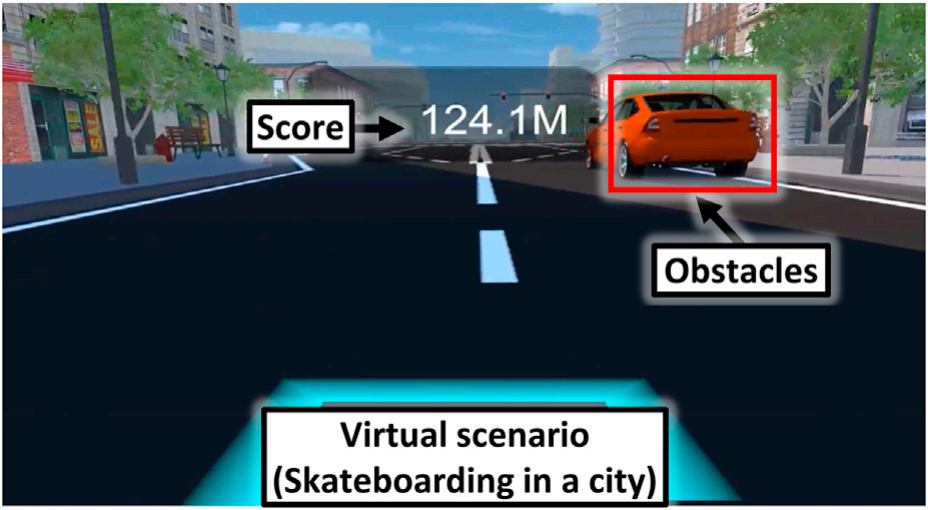
An illustration of a virtual scenario.

A virtual scenario for VR-skateboarding was created using Unity3D software (version 5.3.2, San Francisco, United States) and displayed using virtual reality head-mounted displays (HTC VIVE, HTC Corporation, New Taipei City, Taiwan). The virtual scenario depicted skateboarding on a city road, as shown in Figure 2. Three wireless inertial measurement unit sensors (HTC VIVE trackers, HTC Corporation, New Taipei City, Taiwan) were used in VR-skateboarding as follows: 1) two trackers were placed on the participant’s legs to track leg movements. The speed and distance travelled in the virtual scenario were adjusted based on the movement of the tracker on the moving leg; 2) one tracker was attached in front of the skateboard and used to control the left and right direction of skateboarding to avoid obstacles in the virtual scenario. The cumulative distance travelled was provided as real-time virtual feedback and a final score to motivate participants during VR-skateboarding.

### Participants

The eligibility of participants was assessed based on inclusion and exclusion criteria. The inclusion criteria included being between the ages of 20 and 40 years and not having any symptoms such as leg pain or numbness. The exclusion criteria included having had previous surgery and having neurological disorders such as stroke, lumbar radiculopathy, or spinal cord injury.

### Procedure

For walking, participants were asked to walk on the split-belt treadmill at a comfortable walking speed for 1 min × 5 times. Then, participants were asked to perform VR-skateboarding using their non-dominant leg as the supporting leg on the skateboard. Participants were instructed to skate with their dominant leg as the moving leg at a comfortable walking speed for 1 min × 5 times. According to previous studies, treadmill speed has the potential to impact biomechanical characteristics during movement (Möckel et al., 2003; Matjačić et al., 2019). In order to eliminate confounding factors from treadmill speed, the treadmill was set to the same speed (i.e., comfortable walking speed) for both VR-skateboarding and walking.

## Evaluation

### Joint kinematics measurements

A 3-dimensional motion capture system (VICON ver. 2.5, Oxford Metrics Ltd., Oxford, United Kingdom) with ten infrared cameras (VICON Bonita, Oxford Metrics, United Kingdom) was used to collect joint kinematic data at a sampling rate of 120 Hz. The system used 45 spherical retro-reflective markers (14 mm) placed over anatomical landmarks based on the Plug-In-Gait model (Cheng et al., 202b).

### Muscle activity measurements

Surface electromyography (EMG) (TrignoTM, Delsys Inc., Boston, MA, United States) was used to collect muscle activity data (i.e., erector spinae: trunk extensor; gluteus medius: hip abductor; rectus femoris: knee extensor; and tibialis anterior: ankle dorsiflexor) (Wang et al., 2015). The sampling rate of the EMG was 960 Hz.

### Ground reaction force measurements

Two force platforms (QQ-mill, Motekforce Link, Amsterdam, Netherlands) were used to collect ground reaction force (GRF) data. The force platforms were able to sample at a frequency of 960 Hz using LabVIEW software (National Instruments, Austin, TX, United States).

### Data processing

**FIGURE 3:**
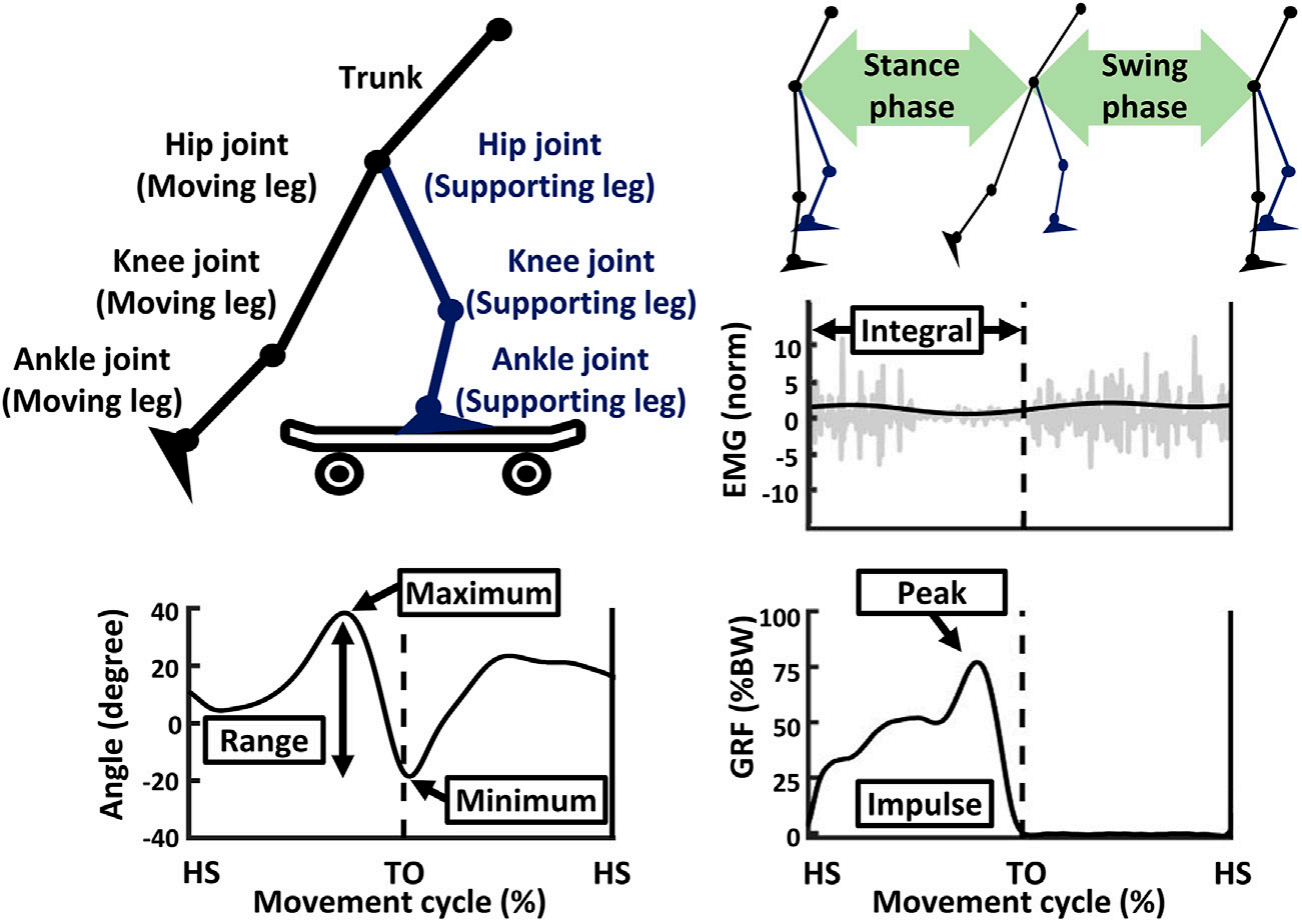
An illustration of data processing. EMG, electromyography; GRF, ground reaction force; %BW, percentage of body weight; HS, heel-strike of moving leg; TO, toe-off of moving leg.

Data from the motion capture system, EMG, and force platforms were processed using a custom program written in MATLAB R2020a software (MathWorks, Natrick, MA, United States). The GRF of the moving leg was used to identify the movement cycle (i.e., the stance and swing phases) of each stride. A GRF threshold of 10 N was used to identify the movement cycle (Baumgart et al., 2017). A total of 100 stable strides were selected for analysis (Kubinski et al., 2015). The stance phase occurs from heel-strike to toe-off, while the swing phase occurs from toe-off to heel-strike, as shown in Figure 3.

For joint kinematics, the data were filtered using a 2nd-order low-pass Butterworth filter with a cut-off frequency of 3 Hz. The filtered data was smoothed using a moving average (Chien and Hsu, 2018). The minimum, maximum, and range values of the trunk, hip, knee, and ankle joints during entire movement cycle were calculated (Smith et al., 2016).

For muscle activities, the EMG data were filtered using a 2nd-order Butterworth filter with bandpass and notch filters at 30–350 Hz and 60 Hz, respectively (Adewuyi et al., 2016). The filtered data was full-wave rectified (using a root mean square) and smoothed (using a moving average) (Tabard-Fougère et al., 2018). The EMG was normalized using the resting EMG for each muscle (Wang et al., 2015). The EMG data was also time-normalized from 0 to 100 percent for each phase (Androwis et al., 2018). The integrals of normalized EMG (i.e., trunk extensor, hip abductor, knee extensor, and ankle dorsiflexor) were then separately reported for the stance and swing phases, as well as for the entire movement cycle (Vigotsky et al., 2017; Wu et al., 2019).

For GRF, the data were filtered using a 2nd-order low-pass Butterworth filter with a cut-off frequency of 5 Hz. The filtered data was smoothed using a moving average (Cheng et al., 2020a). The peak values of GRF in entire movement cycle were computed, while the impulse values of GRF were separately computed for the stance and swing phases, as well as for the entire movement cycle (Golyski et al., 2018; Lee et al., 2020; Jafarnezhadgero et al., 2021).

### Statistical analysis

Statistical analysis was performed in Predictive Analytics Software Statistics 18.0 for Windows (SPSS, Chicago, IL, United States). The normality of all variables was determined using the Shapiro–Wilk test. Nevertheless, the data were not normally distributed. Thus, the non-parametric Wilcoxon signed-rank test was used to compare the variables between VR-skateboarding and walking. The *p*-value was set at 0.05 as statistically significant.

## Results

Twenty young participants (age: 27.4 ± 2.8 years, height: 167.2 ± 10.0 cm, weight: 61.2 ± 11.6 kg, body mass index: 21.7 ± 2.0 kg/m^2^) were recruited, with ten of them being female. All participants were right-leg dominant and used the left leg as the supporting leg and the right leg as the moving leg during VR-skateboarding. The average speed for VR-skateboarding and walking was 1.2 ± 0.1 m/s.

### Joint kinematics

**FIGURE 4:**
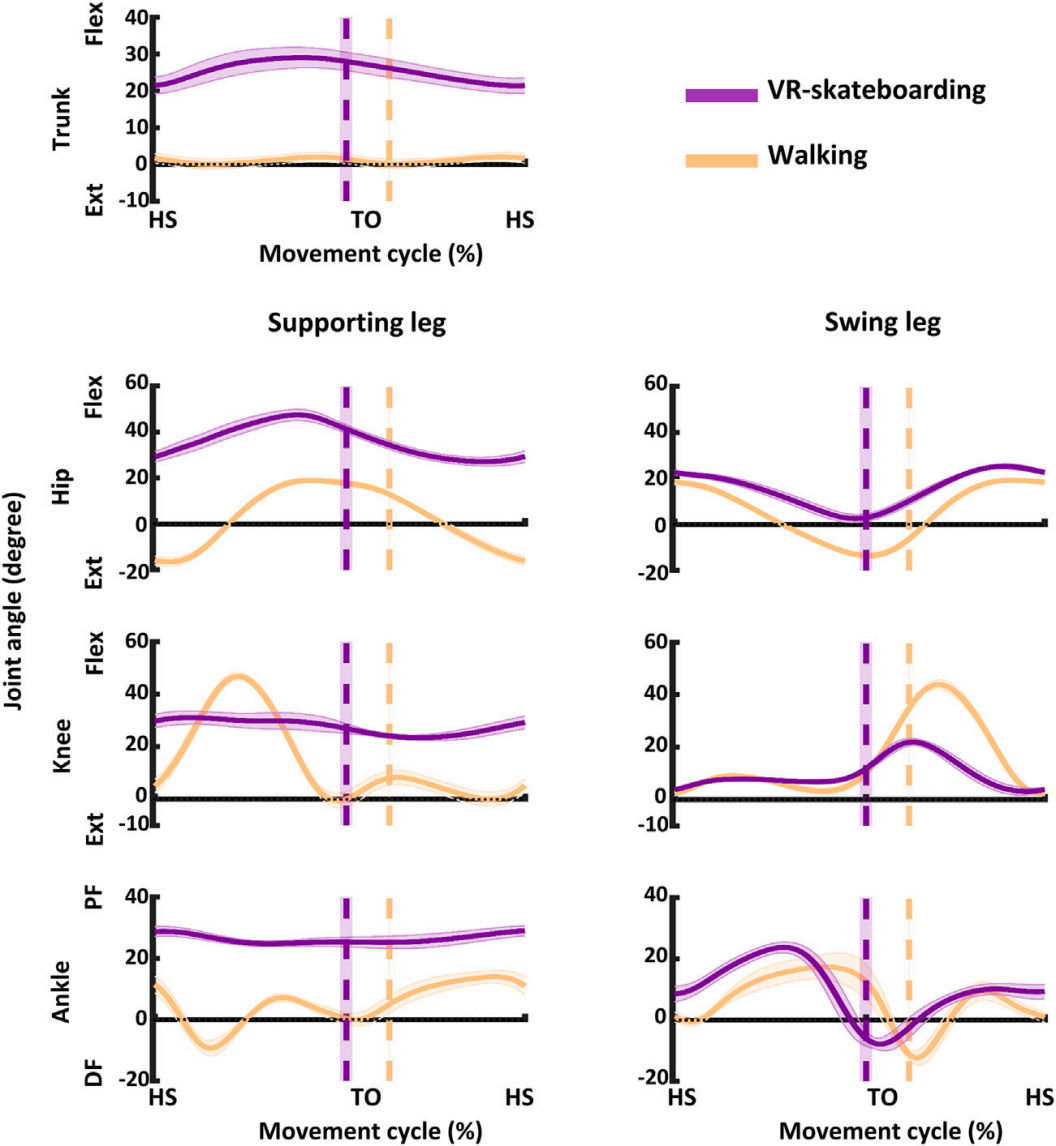
An illustration of joint kinematics. The solid line represents the mean and the shaded area represents the standard deviation. Flex, flexion; Ext, extension; PF, plantarflexion; DF, dorsiflexion; HS, heel-strike of moving leg; TO, toe-off of moving leg; VR-skateboarding, virtual reality skateboarding.

The joint kinematic results for both VR-skateboarding and walking are illustrated in Figure 4; Table 1.

**TABLE 1:** Comparison of the joint kinematics during the entire movement cycle between VR-skateboarding and walking.

Body Segments		Joint Angle (degree)					
		Minimum		Maximum		Range	
		VR-skateboarding	Walking	VR-skateboarding	Walking	VR-skateboarding	Walking
Trunk		20.05 ± 2.14*	−0.45 ± 1.44	30.25 ± 2.60*	2.87 ± 1.25	10.19 ± 2.33*	3.32 ± 1.19
Hip	Supporting Leg	26.02 ± 1.59*	−17.18 ± 2.01	48.32 ± 2.83*	19.30 ± 1.04	22.30 ± 2.23*	36.48 ± 2.24
	Moving Leg	1.80 ± 1.60*	−13.77 ± 1.21	26.16 ± 1.21*	19.85 ± 1.04	24.36 ± 2.22*	33.62 ± 1.39
Knee	Supporting Leg	22.32 ± 1.98*	−1.65 ± 2.39	31.80 ± 3.36*	47.13 ± 1.38	9.48 ± 2.77*	48.78 ± 1.81
	Moving Leg	2.54 ± 1.04*	1.59 ± 1.13	23.28 ± 1.61*	44.99 ± 2.51	20.74 ± 2.00*	43.40 ± 2.24
Ankle	Supporting Leg	24.42 ± 2.79*	−9.03 ± 3.02	30.25 ± 2.12*	15.14 ± 2.53	5.82 ± 2.45*	24.17 ± 2.53
	Moving Leg	−12.09 ± 4.31	−13.08 ± 1.89	27.26 ± 4.84*	18.25 ± 4.50	39.35 ± 7.52*	31.33 ± 4.63

Values are mean ± standard deviation. VR-skateboarding, virtual reality skateboarding. Wilcoxon signed-rank test: *statistically significant values (*p* < 0.05).

During VR-skateboarding, participants exhibited greater minimum and maximum trunk angles and a wider range of movement in their trunk compared to walking (z = −3.92, *p* < 0.01; z = −3.92, *p* < 0.01; and z = −3.92, *p* < 0.01, respectively). This indicated that participants bent their trunk forward more and moved in a wider range during VR-skateboarding when compared to walking.

In the supporting leg, participants demonstrated increased minimum and maximum hip (z = −3.92, *p* < 0.01; and z = −3.92, *p* < 0.01, respectively) and ankle (z = −3.92, *p* < 0.01; and z = −3.92, *p* < 0.01, respectively) angles during VR-skateboarding compared to walking. However, participants had reduced range of movement in the hip and ankle joints (z = −3.92, *p* < 0.01; and z = −3.92, *p* < 0.01, respectively) during VR-skateboarding compared to walking. The knee also showed increased minimum angle (z = −3.92, *p* < 0.01) but reduced maximum angle and range of movement (z = −3.92, *p* < 0.01; and z = −3.92, *p* < 0.01, respectively) during VR-skateboarding. The findings suggest that, in the supporting leg, VR-skateboarding entailed greater flexion in the hip and ankle joints and a smaller range of movement compared to walking. Additionally, during VR-skateboarding, participants demonstrated decreased flexion in the knee joint and a reduced range of movement in this joint.

In the moving leg, the hip joint angles showed a lower range of motion (z = −3.92, *p* < 0.01) during VR-skateboarding compared to walking, with both the minimum and maximum angles being higher (z = −3.92, *p* < 0.01; and z = −3.92, *p* < 0.01, respectively) in VR-skateboarding. The knee joint also showed a lower range of motion during VR-skateboarding (z = −3.92, *p* < 0.01), with the minimum angle being higher (z = −2.72, *p* < 0.01) and the maximum angle being lower (z = −3.92, *p* < 0.01). Whereas, the ankle joint demonstrated a greater range of motion during VR-skateboarding (z = −3.80, *p* < 0.01), with the maximum angle being higher (z = −3.92, *p* < 0.01) and the minimum angle showing no significant difference (z = −1.30, *p* = 0.19) compared to walking. VR-skateboarding involved greater hip flexion and a smaller range of movement in the moving leg compared to walking. However, it also entailed decreased knee flexion and a reduced range of movement, as well as increased ankle dorsiflexion and a greater range of movement.

### Muscle activity

**FIGURE 5:**
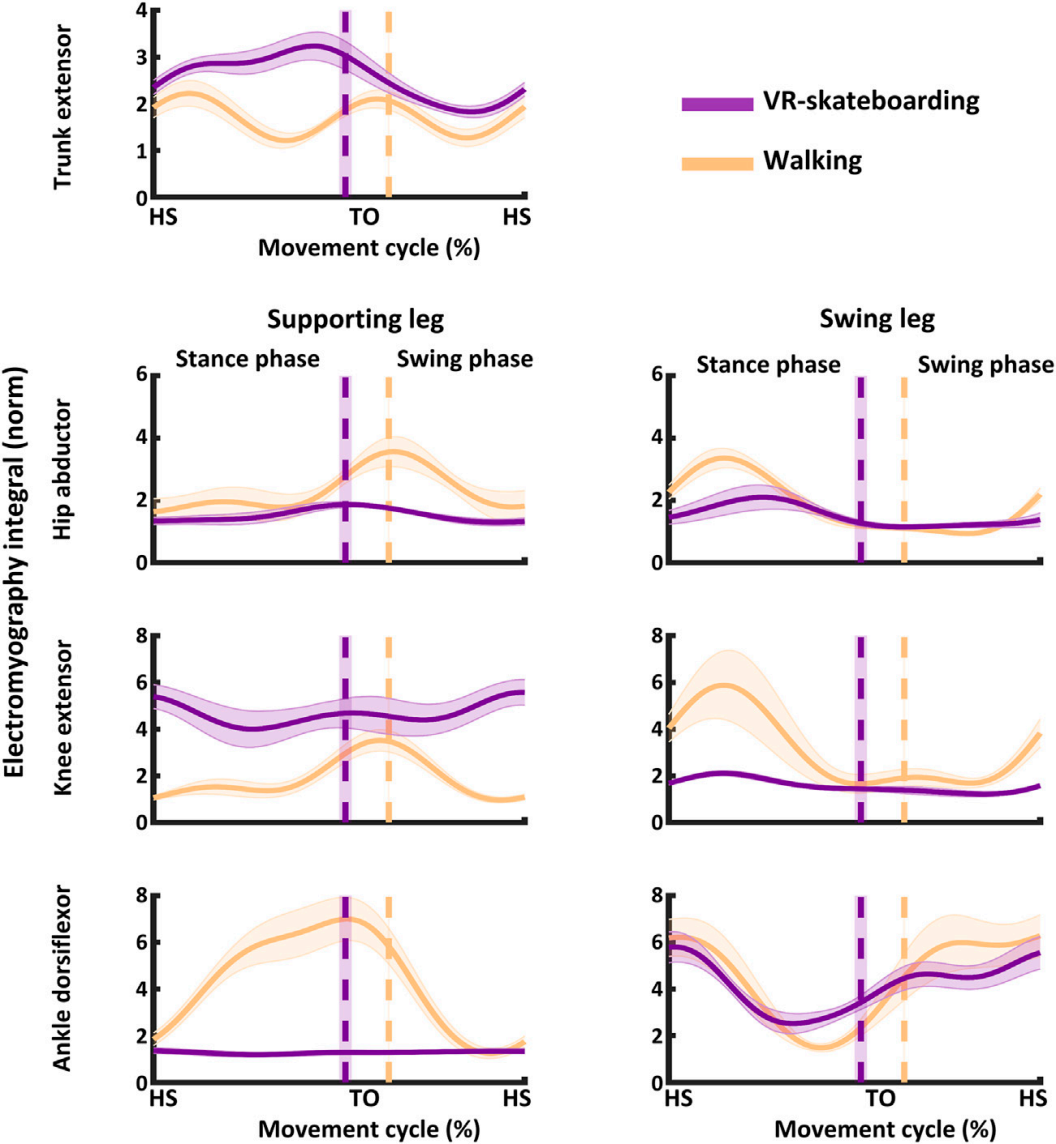
An illustration of muscle activity. The solid line represents the mean and the shaded area represents the standard deviation. HS, heel-strike of moving leg; TO, toe-off of moving leg; VR-skateboarding, virtual reality skateboarding.

The muscle activity results for both VR-skateboarding and walking are illustrated in Figure 5; Table 2.

**TABLE 2:** Comparison of the muscle activity between VR-skateboarding and walking.

Muscle groups		Electromyography integral (norm)					
		Stance Phase		Swing Phase		Entire Movement Cycle	
		VR-skateboarding	Walking	VR-skateboarding	Walking	VR-skateboarding	Walking
Trunk Extensors		2.88 ± 0.23*	1.71 ± 0.21	2.20 ± 0.16*	1.58 ± 0.17	2.54 ± 0.18*	1.65 ± 0.17
Hip Abductors	Supporting Leg	1.49 ± 0.13*	2.19 ± 0.31	1.55 ± 0.09*	2.56 ± 0.26	1.52 ± 0.09*	2.38 ± 0.28
	Moving Leg	1.82 ± 0.45*	2.16 ± 0.17	1.42 ± 0.43*	1.33 ± 0.38	1.62 ± 0.32*	1.75 ± 0.21
Knee Extensors	Supporting Leg	4.10 ± 0.77*	2.57 ± 0.25	4.58 ± 0.53*	2.56 ± 0.26	4.34 ± 0.57*	2.56 ± 0.27
	Moving Leg	1.82 ± 0.18*	3.61 ± 0.79	1.40 ± 0.23*	2.33 ± 0.53	1.61 ± 0.20*	2.97 ± 0.55
Ankle Dorsiflexors	Supporting Leg	1.23 ± 0.11*	2.57 ± 0.24	1.31 ± 0.10*	2.54 ± 0.25	1.27 ± 0.10*	2.56 ± 0.24
	Moving Leg	1.44 ± 0.28*	3.69 ± 0.57	1.42 ± 0.29*	5.92 ± 1.18	1.43 ± 0.27*	4.81 ± 0.79

Values are mean ± standard deviation. VR-skateboarding, virtual reality skateboarding. Wilcoxon signed-rank test: *statistically significant values (*p* < 0.05).

Muscle activity of the trunk extensor in the stance phase, swing phase, and entire movement cycle was higher during VR skateboarding than during walking (z = −3.92, *p* < 0.01; z = −3.92, *p* < 0.01; and z = −3.92, *p* < 0.01, respectively). This indicated that VR-skateboarding appeared to involve higher muscle activity in the trunk extensor muscles compared to walking.

In the supporting leg, muscle activity of the knee extensor was higher in the stance phase, swing phase, and entire movement cycle (z = −3.92, *p* < 0.01; z = −3.92, *p* < 0.01; z = −3.92, *p* < 0.01; and z = −3.92, *p* < 0.01, respectively) during VR-skateboarding than during walking. However, muscle activity in the hip abductor and ankle dorsiflexor was lower in the stance phase (z = −3.80, *p* < 0.01; and z = −3.92, *p* < 0.01, respectively), swing phase (z = −3.92, *p* < 0.01; and z = −3.92, *p* < 0.01, respectively), and entire movement cycle (z = −3.92, *p* < 0.01; and z = −3.92, *p* < 0.01, respectively) during VR-skateboarding than during walking. The results suggest that VR-skateboarding entailed distinct muscle activity patterns compared to walking, specifically higher activity in the knee extensor but lower activity in the hip abductor and ankle dorsiflexor.

In the moving leg, VR-skateboarding was associated with lower muscle activity in the hip abductor during the stance phase (z = −3.17, *p* < 0.01) and entire movement cycle (z = −2.68, *p* < 0.01) compared to walking, but no significant difference was observed in the swing phase (z = −0.85, *p* = 0.39). Additionally, VR-skateboarding involved lower muscle activity in the knee extensor and ankle dorsiflexor in the stance phase (z = −3.92, *p* < 0.01; and z = −3.92, *p* < 0.01, respectively), swing phase (z = −3.92, *p* < 0.01; and z = −3.92, *p* < 0.01, respectively), and entire movement cycle (z = −3.92, *p* < 0.01; and z = −3.92, *p* < 0.01, respectively) compared to walking. These findings suggest that muscle activity in the hip abductor, knee extensor, and ankle dorsiflexor of the moving leg was lower during VR-skateboarding compared to walking, except for the hip abductor in the swing phase, which showed no difference.

### Ground reaction force

**FIGURE 6:**
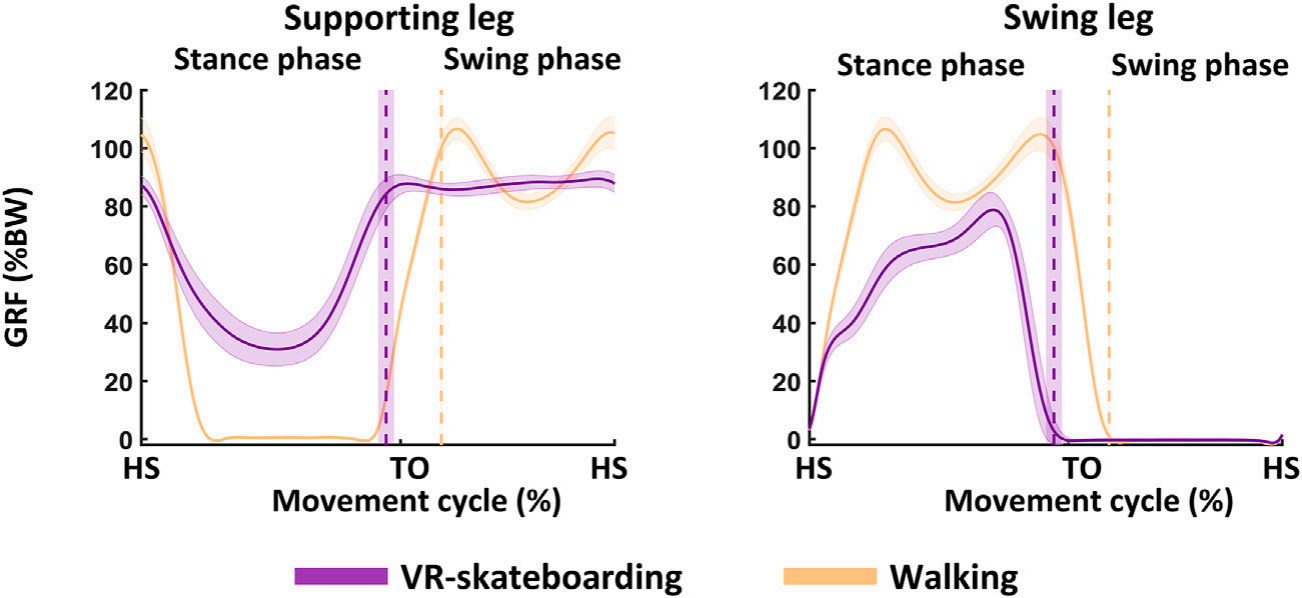
An illustration of ground reaction force. The solid line represents the mean and the shaded area represents the standard deviation. GRF, ground reaction force; %BW, percentage of body weight; HS, heel-strike of moving leg; TO, toe-off of moving leg; VR-skateboarding, virtual reality skateboarding.

Our results showed that the average stance phase during walking was 63.58% ± 0.24%. During VR-skateboarding, the average stance phase was 51.91% ± 1.74%. Hence, the stance phase was shorter during VR-skateboarding than during walking (z = −3.92, *p* < 0.01). The GRF results for both VR-skateboarding and walking are illustrated in Figure 6; Table 3 and Table 4.

**TABLE 3:** Comparison of the peak ground reaction force during the entire movement cycle between VR-skateboarding and walking.

Body segments	Peak ground reaction force (% body weight)	
	VR-skateboarding	Walking
Supporting leg	95.64 ± 2.08*	111.76 ± 2.35
Moving leg	83.28 ± 5.39*	111.48 ± 2.71

Values are mean ± standard deviation. VR-skateboarding, virtual reality skateboarding. Wilcoxon signed-rank test: *statistically significant values (*p* < 0.05).

**TABLE 4:** Comparison of the impulse ground reaction force between VR-skateboarding and walking.

*Body Segments*	*Impulse Ground Reaction Force (% Body Weight)*					
	Stance phase		Swing phase		Entire movement cycle	
	VR-skateboarding	Walking	VR-skateboarding	Walking	VR-skateboarding	Walking
Supporting leg	48.34 ± 5.84*	24.79 ± 0.85	87.95 ± 2.20*	93.64 ± 3.08	68.15 ± 3.05*	59.21 ± 1.88
Moving leg	53.51 ± 3.88*	76.72 ± 3.22	N/A	N/A	N/A	N/A

Values are mean ± standard deviation. VR-skateboarding, virtual reality skateboarding; N/A, not applicable. Wilcoxon signed-rank test: *statistically significant values (*p* < 0.05).

In the supporting leg, the peak GRF during VR-skateboarding was lower than during walking (z = −3.92, *p* < 0.01). However, the impulse GRF during VR skateboarding was higher in the stance phase (z = −3.92, *p* < 0.01) and entire movement cycle (z = −3.92, *p* < 0.01) but lower in the swing phase (z = −3.92, *p* < 0.01) compared to walking. These results indicated that during VR-skateboarding, the force loading on the supporting leg and weight distribution in the swing phase were less compared to walking, but there was a greater distribution of weight during the stance phase and throughout the entire movement cycle.

In the moving leg, the peak and impulse GRF during VR-skateboarding was lower than walking (z = −3.92, *p* < 0.01; and z = −3.92, *p* < 0.01, respectively). The results showed that during VR-skateboarding, the force loading on the moving leg and weight distribution in the swing phase were lower compared to walking.

## Discussion

Our study found that there were differences in joint kinematics, muscle activity, and weight distribution between VR-skateboarding and walking, particularly in the supporting leg. Previous research on skateboarding had primarily focused on the “ollie” technique, which involves using the skateboard to jump over obstacles, and therefore was not directly comparable to VR-skateboarding in our study (Frederick et al., 2006; Hu et al., 2021). Our results indicated that the supporting leg during VR-skateboarding involved higher trunk, hip, and ankle movements, as well as higher muscle activity of the knee extensor, and a higher weight distribution compared to walking. Based on these findings, we recommended VR-skateboarding as a potential rehabilitation training approach for improving balance.

During VR-skateboarding, participants demonstrated a greater range of trunk movement and flexion compared to walking. Previous research has indicated that trunk bending can help maintain the center of mass within the base of support during activities such as a unilateral squat (Eliassen et al., 2018; van den Tillaar and Larsen, 2020). In our study, VR-skateboarding involved balancing on a skateboard, which may have required participants to lean forward or bend their trunk to maintain balance and control. This may have explained the observed increase in trunk flexion during VR-skateboarding, as such movements may not have been necessary for walking. Our findings were consistent with previous studies that have shown that increases in trunk flexion can enhance muscle activity in the trunk extensor muscles eccentrically during the stance and swing phases (Voglar et al., 2016; Yoder et al., 2019). The flexion position involved in VR-skateboarding may also have increased the demands on the trunk extensor to maintain balance and control. Additionally, it is possible that the use of VR technology in VR-skateboarding may have contributed to the observed differences in trunk angles and movement (Horsak  et al., 2021; Meinke et al., 2022). The visual input provided by the VR headset may have influenced the participant’s trunk angles and movement in order to maintain balance and control within the virtual environment (Lin et al., 2019a; Benady et al., 2021). As a result, the increased trunk bending during VR-skateboarding may have led to higher muscle activity in the trunk extensor compared to walking. The increased trunk bending during VR-skateboarding leading to higher muscle activity in the trunk extensor may be an effective approach of exercising to improve balance.

In the supporting leg, VR-skateboarding resulted in higher hip and ankle joint kinematics, as well as increased muscle activity of the knee extensor, compared to walking. This could be attributed to the height difference between the skateboard and the treadmill belt, which required participants to constantly flex the joints in the supporting leg to maintain balance. Previous research has shown that the knee extensor and hip abductor in the supporting leg are activated to hold body weight during unilateral squatting (Eliassen et al., 2018; van den Tillaar and Larsen, 2020). Our study also found that the activation of the knee extensor in the supporting leg during VR-skateboarding was higher than during walking, both in the stance and swing phases. However, the activation of the hip abductor was lower during VR-skateboarding, possibly due to the support provided by the handrail (Komisar et al., 2019). In addition, the activation of the ankle dorsiflexor was lower during VR-skateboarding due to the stationary position of the supporting foot. This fixed position of the ankle joint may also have contributed to the decrease in ankle dorsiflexor activation observed in previous studies (Macrum et al., 2012; Guillén-Rogel et al., 2017).

In the moving leg, VR-skateboarding required participants to increase hip flexion and ankle dorsiflexion in order to maintain trunk flexion and clear their feet from the ground. However, participants exhibited lower joint angles of knee flexion during VR-skateboarding compared to walking. This may have been due to the shorter stance phase in VR-skateboarding, which can limit the range of motion at the hip and knee joints. Previous research has shown that a shorter stance phase can result in reduced knee flexion angles (Yen et al., 2019). Additionally, the muscle activity of the lower extremity in the non-weight-bearing leg tends to decrease when body weight is reduced or shifted to the other leg (Clark et al., 2004; Kristiansen et al., 2019). Our results showed a decrease in muscle activity of the hip abductor and knee extensor in the moving leg, which may have been a result of participants shifting their weight to the supporting leg. However, while the joint kinematics and muscle activation in the moving leg were reduced, the weight shifting to the supporting leg can be beneficial for balance training of the unilateral leg specifically.

The results of this study indicated that VR-skateboarding was associated with a lower force loading but higher weight distribution on the supporting leg when compared to walking. This decrease in force loading was believed to be due to the support provided by holding onto the handrail, while the increase in weight distribution was likely due to weight shifting (Clark et al., 2004; Wu et al., 2018; Kristiansen et al., 2019). Weight shifting has been found to improve balance by strengthening muscles, improving coordination, and enhancing control of the body’s center of mass, leading to increased stability during movement (Lin et al., 2019a; Lin et al., 2019b). Additionally, VR-skateboarding was found to have a shorter stance phase for the moving leg compared to walking. Although this study conducted VR-skateboarding at the same speed as walking, there were still differences in movement cycles. One possible explanation for this is that both legs were moving during walking, so participants needed to shift their center of mass to the new base of support provided by the supporting leg (Lin et al., 2019a). This process required time and distance to complete (Lin et al., 2019a). However, in VR-skateboarding, the supporting leg consistently supported the body weight, allowing the moving leg to swing more freely. Previous research has shown that gait training with a shorter stance phase can reorganize walking patterns and improve walking speed (Stolze et al., 2001; Yen et al., 2019). The decreased force loading, increased weight distribution, and reduced duration of movement cycles observed in VR-skateboarding may have the potential to reduce joint loading, enhance weight bearing, and improve walking speed, respectively. These factors may have contributed to the potential benefits of VR-skateboarding as a rehabilitation tool for individuals with balance impairments.

### Clinical implications

The clinical significance of this study is the potential use of VR-skateboarding as a rehabilitation training approach to improve balance. When performing VR-skateboarding, participants had greater range of movement and flexion in their trunk compared to when walking, as well as increased muscle activity and weight distribution in the supporting leg. These differences in biomechanics contributed to increase joint and muscle coordination during VR-skateboarding. Additionally, VR-skateboarding might also be considered a type of closed kinetic chain exercise, where the foot is fixed. Our results indicated that participants consistently kept the foot of the supporting leg on the skateboard. Previous studies have revealed that closed kinetic chain exercises can promote muscle co-contraction to stabilize the trunk and legs. This muscle co-contraction also aids in improving proprioception, or the ability to sense the position and movement of one’s body in space, subsequently improving balance. Furthermore, the use of VR technology may have influenced the observed differences in joint kinematics, muscle activity, and weight distribution. These findings suggest that VR-skateboarding may be an effective training approach for improving balance in individuals undergoing rehabilitation.

In addition, the consideration of the biomechanical characteristics of VR-skateboarding is important for software engineers creating new features and implementing multidisciplinary approaches. By understanding the biomechanics, software engineers can design features that are ergonomic and user-friendly, ensuring that the products they develop are comfortable, safe, and appropriate for balance training. This is particularly important for products or systems that would be used by a wide range of individuals with different physical abilities and characteristics, as it allows for the creation of solutions that are accessible and inclusive. Incorporating a multidisciplinary approach also enables software engineers to consider the diverse needs and perspectives of different populations, such as those with unilateral leg symptoms, to create well-rounded and effective solutions. Overall, the incorporation of biomechanical characteristics in the development process can lead to the creation of innovative and highly functional products and systems that meet the needs of a diverse range of users.

### Study limitations

This study had a few limitations. First, for safety reasons, participants held onto a handrail while VR-skateboarding. This may have partially supported their body weight and potentially affected joint kinematics, muscle activity or weight distribution. However, we believed that handrail use is necessary in patient populations to prevent accidents during training. Second, this study conducted the experiment on the same population and at the same speed (i.e., comfortable walking speed). Moreover, participants were all healthy individuals with no leg abnormalities. Therefore, the results of VR-skateboarding in this study should be interpreted with caution when applied to patient populations or different speeds. Third, participants performed VR-skateboarding and walking with bare feet in this study. According to previous studies, wearing shoes can change biomechanical characteristics, particularly reducing force loading (Zhang et al., 2013; Udofa et al., 2019). Hence, this factor should be taken into consideration when applying our findings to VR-skateboarding while wearing shoes.

## Conclusion

VR-skateboarding was a novel VR-based balance training approach. The results of our study demonstrated that VR-skateboarding involved increased movement and muscle activity in the trunk, hips, and ankles, particularly in the supporting leg, compared to walking. The weight distribution was also found to increase when participants stood on the skateboard with their supporting leg. These findings suggested that VR-skateboarding may be a promising rehabilitation tool for improving balance. Additionally, we proposed that future developments and applications of this training should prioritize the strengthening of the supporting leg in order to maximize its therapeutic benefits.

## Data Availability

The raw data supporting the conclusion of this article will be made available by the authors, without undue reservation.
